# Supplementary material for: Exploring the Binding of Natural Compounds to Cancer-Related G-Quadruplex Structures: From 9,10-Dihydrophenanthrenes to Their Dimeric and Glucoside Derivatives
Source: Int J Mol Sci. 2023 Apr 24;24(9):7765. doi: 10.3390/ijms24097765 (PMC10178421; doi:10.3390/ijms24097765)
Supplement: Supplementary file 1 [file ijms-24-07765-s001.zip › ijms-2333460-supplementary.pdf]

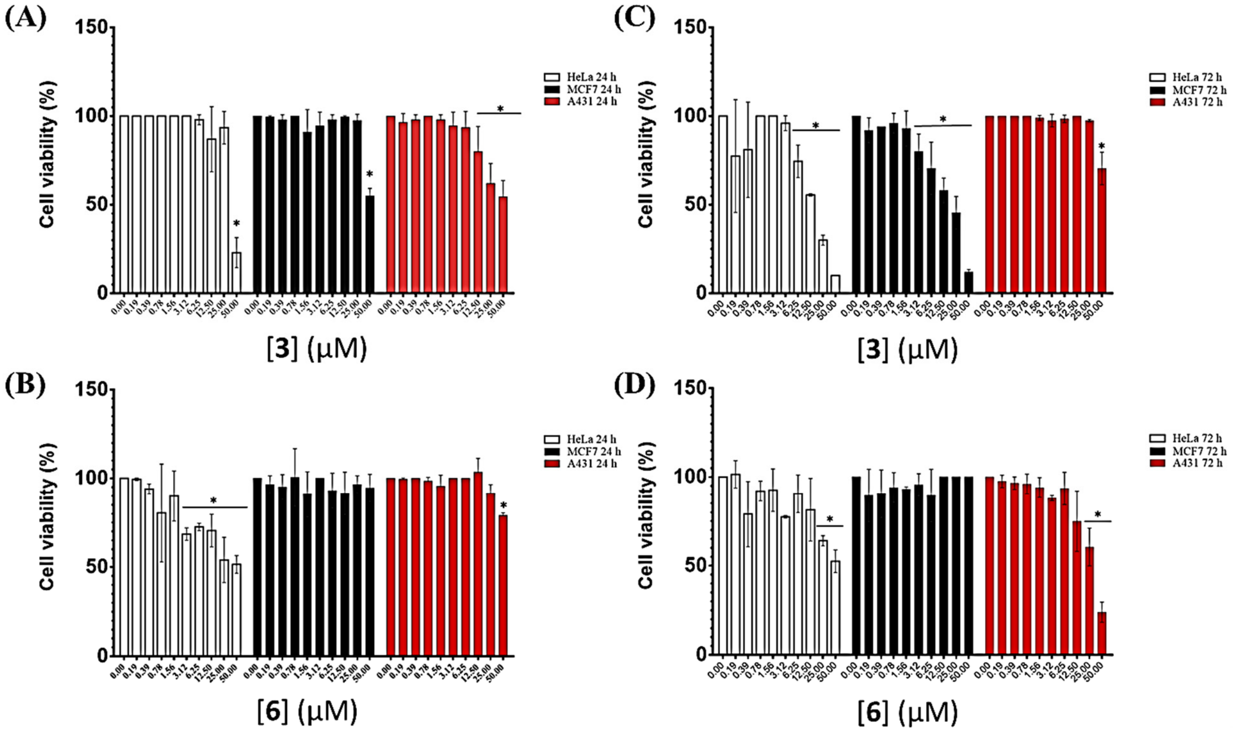

Figure S1. Effects of increasing concentrations of **3** and **6** (0-50 μM) on the viability of HeLa, MCF7 and A431 human cancer cells upon 24 h (A and B, respectively) and 72 h (C and D, respectively) of incubation. Cell viability values are expressed as the percentage of viable cells for treated *vs.* control cells grown in the absence of the tested compounds. Three independent experiments were performed with triplicated determinations. *P < 0.05 values were obtained for treated *vs.* control samples.
